# Every Coin Has a Back Side: Invasion by *Limnohabitans planktonicus* Promotes the Maintenance of Species Diversity in Bacterial Communities

**DOI:** 10.1371/journal.pone.0051576

**Published:** 2012-12-12

**Authors:** Karel Horňák, Gianluca Corno

**Affiliations:** 1 Biology Centre of the Academy of Sciences of the Czech Republic, v.v.i., Institute of Hydrobiology, České Budějovice, Czech Republic; 2 Limnological Station, Institute of Plant Biology, University of Zürich, Kilchberg, Switzerland; 3 CNR – Institute of Ecosystem Study, Verbania, Italy; University of Delaware, United States of America

## Abstract

One of the earliest challenges for ecologists has been to study the impact of invasive species on microbial communities. Although bacteria are fundamental in biological processes, current knowledge on invasion effects by aquatic non-pathogenic bacteria is still limited. Using pure cultures of diverse planktonic bacteria as model organisms at two different carbon concentration levels, we tested the response of an assembled community to the invasion by *Limnohabitans planktonicus*, an opportunistic bacterium, successful in freshwaters. The invader, introduced at the early stationary growth phase of the resident community, caused a strong decrement of the abundance of the dominant species. This was due to competition for nutrients and a potential allelopathic interaction. Simultaneously, resident species formerly unable to successfully compete within the community, thus potentially exposed to competitive exclusion, increased their abundances. The overall result of the invasion was preservation of species diversity, the higher the lower was the substrate content available. Our study provides new insights into bacterial invasions, offering an alternative interpretation of invasions for community ecology.

## Introduction

Microbial invasions can drastically affect microbial community structure and functioning, and in turn also the regulation of primary productivity and of every biogeochemical cycle on a world scale [1]. Although a number of studies have focused on the impact of microbial invasions on plants and animals [2], or on the ecological factors driving pathogens diversity and distribution [3], little is known about the impact of invasive non-pathogenic bacteria in aquatic environments. Moreover, microbial invasions are generally reported in terms of negative effects on the resident community and ecosystem such as: how exotic species can outcompete native ones, how they can alter or reduce established interactions, to which extend they facilitate further invasions [4], [5].

However, invasive organisms named “ecosystem engineer species” [6] may have positive effects on the stability and diversity of the invaded habitat by promoting new ecological niches and interactions [7] or by modifying the community consumption rates on available resources [8]. The potential success of an invasion and the magnitude of its impact on the resident community are influenced by dispersal rates of the invader and by the local environment (i.e. species sorting) [9], [10]. Positive impacts of invasions on diversity and productivity of a system have been attributed to a variety of organisms invading a broad range of habitats, including aquatic ecosystems [7], [11], always characterized by very defined native communities [12], [13]. More studies possibly using known bacterial species to obtain realistic models are essential to understand microbial invasions and their prediction [1].

Here, we experimentally tested the response of a simplified community of sympatric species isolated from a large European lake to an invasion by another bacterial species, itself typical for freshwater habitats. We tested the growth and competitiveness of bacteria in artificial communities (mixed co-cultures) under two contrasting levels of available substrates using *Arthrobacter agilis* strain GC027, *Aeromonas hydrophila* strain GC015, *Brevundimonas* sp. strain GC044, and *Flavobacterium* sp. strain 16mp4. These four species were reckoned to be suitable for the experiment since they (i) represent well-defined members of the major phylogenetic groups of planktonic bacteria, evolutionary divergent from each other; (ii) possess different lifestyles and morphologies [14]; (iii) are easily cultivable in standard cultivation media. Growth patterns of the strains achieved in the communities were compared to those in pure culture. Subsequently, we examined the invasion impact by the competitive and opportunistic bacterium *Limnohabitans planktonicus* strain II-D5^T^ [15] on the established communities at the early stationary growth phase. *L. planktonicus* was also selected because of its ecophysiological traits, that make this species an ideal invasive microbe [16]: it is common and generally abundant in freshwaters [17], with high growth and substrate uptake rates [18], [19], being limited in nature by protistan predation [20], [21]. Specifically, we tested whether the invasion altered species diversity and community structure.

## Materials and Methods

### Bacterial Strains

*Limnohabitans planktonicus* strain II-D5^T^ [15] is a Gram-negative, fast-growing, non-motile, rod-shaped bacterium with a free-living planktonic lifestyle affiliated with the genus *Limnohabitans* [22] within the family *Comamonadaceae* (*Betaproteobacteria*). The strain was isolated from the surface layer of the freshwater meso-eutrophic Římov reservoir (Czech Republic). *L. planktonicus* is common and abundant particularly in non-acidic freshwater habitats including European lakes [17], where it is generally limited by high predation pressure by heterotrophic flagellates, main bacterial predators in aquatic ecosystems [20], [21]. A partial sequence of the 16S rRNA gene of *L. planktonicus* has been deposited in the GenBank (accession number FM165535).

*Arthrobacter agilis* strain GC027, *Brevundimonas* sp. strain GC044 and *Aeromonas hydrophila* strain GC015 were isolated from an enrichment culture from Lake Zürich in autumn 2009 by flow-cytometric single cell sorting and subsequent re-growth of the pure cultures in ALW medium (further details in experimental setup). *A. agilis* strain GC027 and *Brevundimonas* sp. strain GC044, were identified by partial sequencing of their 16Sr RNA genes (deposited in the GenBank under accession numbers JN009621 and JN009622). The Gram-positive *A. agilis* strain GC027 (*Actinobacteria*) is a well-known plant pathogen and can be found in freshwaters only occasionally [23] as it is limited by its highly reduced ability in substrate uptake. Members of the genus *Brevundimonas* (*Alphaproteobacteria*, Gram-negative) are common in freshwaters where some can adapt to low carbon conditions [14]. *Brevundimonas* sp. strain GC044 is highly limited by predation in co-cultures with flagellates [G. Corno, unpublished data]. *A. hydrophila* (*Gammaproteobacteria*) is a Gram-negative rod, motile, and facultative anaerobe, common in freshwaters [24] with chitinolytic properties [25]. Its fast generation time limits the impact of flagellate predation during phytoplankton blooms (G. Corno, unpublished data). *Flavobacterium* sp. strain 16mp4 (FLAV2, *Bacteroidetes*, partial 16Sr RNA gene deposited in GenBank under accession number FN179350) was also isolated from an enrichment culture from Lake Zürich in 2006 [26]. It was selected as a member of our resident community because of its particular lifestyle: even being rather common in lakes it is almost always limited to very small populations, but it is stimulated by algal exudates during the spring phytoplankton bloom [26] and it has been shown to readily incorporate N-acetyl-glucosamine (NAG) [27]. *L. planktonicus*, *A. hydrophila*, and *Brevundimonas* can be regarded as members of the “Abundant Biosphere” in lakes, comprised by a few tens of the core microbial species that regularly form large populations in a specific habitat [28]. Our resident community was thus composed of a mixture of species with different ecophysiological capabilities, and with different ecological success in waters, in order to reproduce a very simplified natural environment. All the strains used in the study are further referred to as *A. agilis*, *A. hydrophila, Brevundimonas*, *Flavobacterium*, and *L. planktonicus.* No specific permits were required for the described field studies.

### Experimental Setup

Prior to the experiment, *A. agilis*, *A. hydrophila, Brevundimonas*, *Flavobacterium*, and *L. planktonicus* were separately pre-cultivated for 3 days in the dark at 20°C in inorganic artificial lake water (ALW) medium [29]. Equal amounts of peptone, yeast extracts, and glucose were added yielding a low- (LCC) and a high-carbon concentration (HCC) treatment in which the total concentration of organic supplements was 9 and 45 mg C l^−1^, respectively. Total abundances of bacteria were determined by epifluorescence microscopy. The preconditioned strains were subsequently inoculated into 160 ml of fresh ALW in 250 ml Erlenmeyer flasks to initial cell abundances of approximately 1×10^6^ cell ml^−1^. Pure cultures of *A. agilis*, *A. hydrophila, Brevundimonas*, *Flavobacterium*, and *L. planktonicus* as well as mixed co-cultures consisting of equal abundances of *A. agilis*, *A. hydrophila, Brevundimonas*, and *Flavobacterium* were grown in triplicates for 168 h at 20°C in the dark (subsequently referred to as control community). In parallel, a replicate for each co-culture (in total three replicates for each carbon concentration) was additionally amended with *L. planktonicus* (initial abundance corresponded to ∼1% of total abundance of resident bacteria) at 72 h of cultivation (subsequently referred to as invaded community). Temporal changes in growth patterns of each strain achieved in (i) control community vs. pure cultures and in (ii) invaded community vs. control community were compared. Subsamples for bacterial abundances and catalyzed reporter deposition-fluorescence *in situ* hybridization (CARD-FISH) were taken daily between 72–168 h of incubation.

### Abundance of Bacteria

Samples for the determination of bacterial abundance were fixed with freshly prepared buffered paraformaldehyde (PFA) (final concentration, 1%). Bacterial strains were stained with 4′,6-diamidino-2-phenylindole (DAPI) (final concentration, 1 µg l^−1^) and concentrated on 0.2 µm polycarbonate filters (type GE Black, diameter 25 mm, Whatman). Bacterial abundances were enumerated using epifluorescence microscopy (Axioskop, Zeiss, Germany). At least 500 cells were enumerated per sample.

### Community Composition and Diversity

The composition of the bacterial co-cultures was analysed by CARD-FISH. Firstly, bacterial strains from co-cultures were fixed with freshly prepared buffered PFA (final concentration, 1%) and subsequently concentrated on 0.2 µm polycarbonate filters (type GTTP, diameter 25 mm, Millipore). Filters were rinsed twice with sterile phosphate buffered saline (PBS), air-dried and stored at −20°C until further processed. The following horseradish peroxidase-labeled probes were used to determine the relative proportions of specific bacterial populations: ALF968 for *Alphaproteobacteria* [30], BET42a for *Betaproteobacteria* [30] and GAM42a for *Gammaproteobacteria* [31] both mixed with the corresponding competitor probe, CF319a for *Cytophaga-Flavobacteria* [32], and HGC69a for *Actinobacteria* [33]. CARD-FISH was performed according to Sekar and co-workers [34]. Subsequently, bacteria were stained with DAPI (final concentration 1 µg l^−1^) and the relative proportions of hybridized cells were determined by epifluorescence microscopy using a fully automated epifluorescence microscope (AxioImager.Z1, Zeiss, Germany) equipped with a motorized stage for microscopic slides, a LED epifluorescence illumination device (Colibri, Zeiss) and the filter set 62 HE (Zeiss). Imaging was performed using a CCD camera (AxioCam MRm, Zeiss) and the image analysis software AxioVision 4.6 (Zeiss). At least 500 cells were counted per sample. In all samples, the overall CARD-FISH detection rate was 90–100% of total bacterial number (mean 95.1%, standard deviation 5.3). Overall community diversity in different treatments was estimated by applying the Shannon-Wiener diversity index (*H’*) calculated by using single species abundance for each sample. *H’* in invaded communities was determined without regards of the relative contribution of *L. planktonicus*. Due to low variability in FISH-detection rates, only a minimal effect on the overall trend in *H’* was assumed.

### Statistical Analyses

A two-way repeated measures ANOVA was applied to test for significant differences in: (i) abundances of bacterial strains or in (ii) species diversity between invaded and control communities, respectively. All statistics were performed using GraphPad Prism (GraphPad Software, USA).

## Results

### Bacterial Growth Patterns

**Figure 1:**
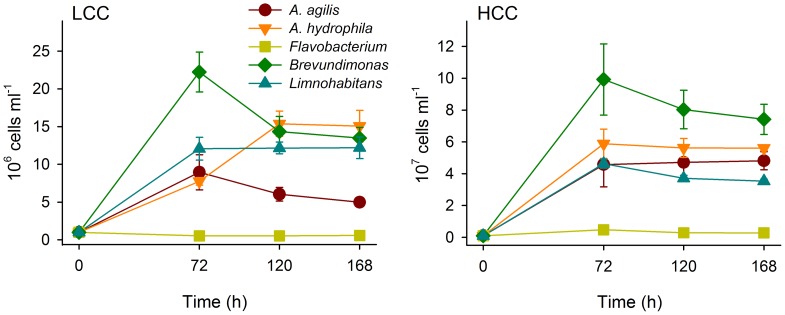
Bacterial growth in pure cultures. Time-course changes in abundance of *A. agilis*, *A. hydrophila, Brevundimonas* sp., *Flavobacterium* sp., and *L. planktonicus* (invader) grown in pure cultures either in low- (LCC) or in high-carbon concentration (HCC) treatments. Note different scales of y-axes in different panels. Values are means of triplicates, error bars = SD.

In pure cultures, all bacterial strains apart from *Flavobacterium* revealed pronounced growth but with different magnitudes and temporal patterns in low- (LCC) and high-carbon concentration (HCC) treatments (Figure 1). *Brevundimonas* achieved highest abundances at 72 h of incubation in both LCC and HCC treatments followed by a decrease towards the end of the experiment (Figure 1). In contrast, abundances of *Flavobacterium* remained virtually identical over the whole cultivation period in both treatments. In comparison, *L. planktonicus*, *A. agilis*, and *A. hydrophila* achieved intermediate abundances at 72 h in both LCC and HCC treatments. While abundances of *A. hydrophila* further increased in LCC treatment the opposite was found for *A. agilis* (Figure 1). In HCC treatment, abundances of *L. planktonicus* further decreased compared to constant abundances of *A. hydrophila* and *A. agilis* between 72 and 168 h of incubation (Figure 1).

**Figure 2:**
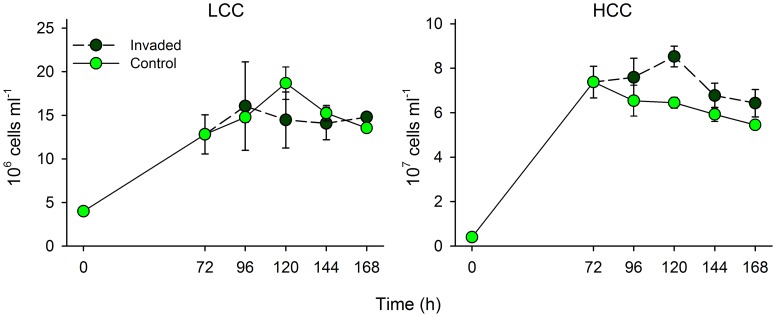
Community growth. Temporal changes in abundances of total bacteria grown in control community or in the community invaded by *L. planktonicus* either in low- (LCC) or in high-carbon concentration (HCC) treatments. Note different scales of y-axes in different panels. Values are means of triplicates, error bars = SD.

**Figure 3:**
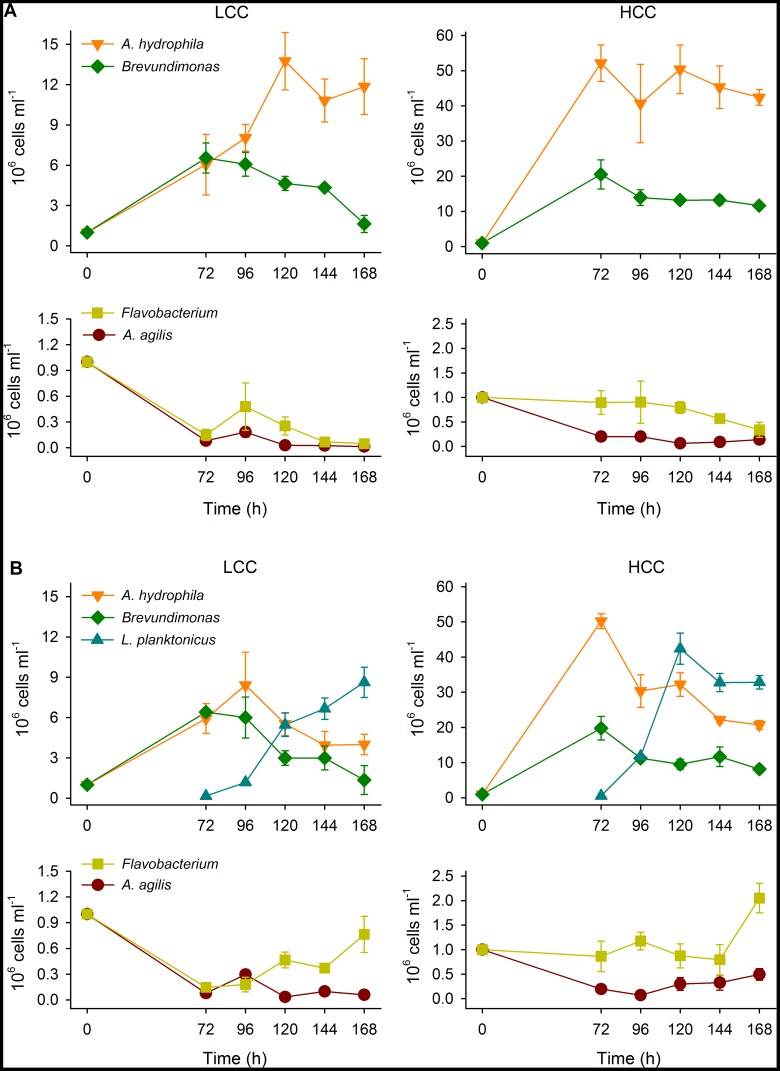
Bacterial growth in communities. Temporal changes in abundances of *A. agilis*, *A. hydrophila, Brevundimonas* sp., *Flavobacterium* sp., grown in control community (A) or in the community invaded by *L. planktonicus* (B) either in low- (LCC) or in high-carbon concentration (HCC) treatments. Note different scales of y-axes in different panels. Values are means of triplicates, error bars = SD.

Total bacterial abundances in co-cultures rapidly increased in both LCC and HCC treatments within 72 h and remained stable or further decreased in LCC and HCC treatment, respectively (Figure 2). No significant differences in total bacterial abundances between control and invaded communities were observed in all but HCC treatment at 120 h (two-way repeated measures ANOVA, P<0.05, n = 6). Control communities that were grown for 3 days were dominated by both *Brevundimonas* and *A. hydrophila* in the LCC treatment while the latter strain clearly dominated the community in the HCC treatment (Figure 3A). Abundances of *A. hydrophila* further increased in LCC or were stable in HCC whereas those of *Brevundimonas* constantly decreased towards the end of the experiment in both treatments. Both *A. agilis* and *Flavobacterium* constituted only negligible proportions in the LCC and HCC treatments during the experiment with abundances lower than their initial abundances (Figure 3A).

### Invasion Effects on Community Composition and Diversity

After inoculation at 72 h, *L. planktonicus* was able to successfully invade the resident communities and it became the most abundant component in both LCC and HCC communities after 120 and 96 h (Figure 3B), respectively. Abundances of *A. hydrophila* that formerly dominated the communities decreased towards the end of incubation. *Brevundimonas* revealed very similar temporal changes to those in control community. Interestingly, the abundance of *Flavobacterium* increased towards the end of the experiment in both the LCC and HCC communities while those of *A. agilis* increased in the HCC treatment only (Figure 3B).

**Figure 4:**
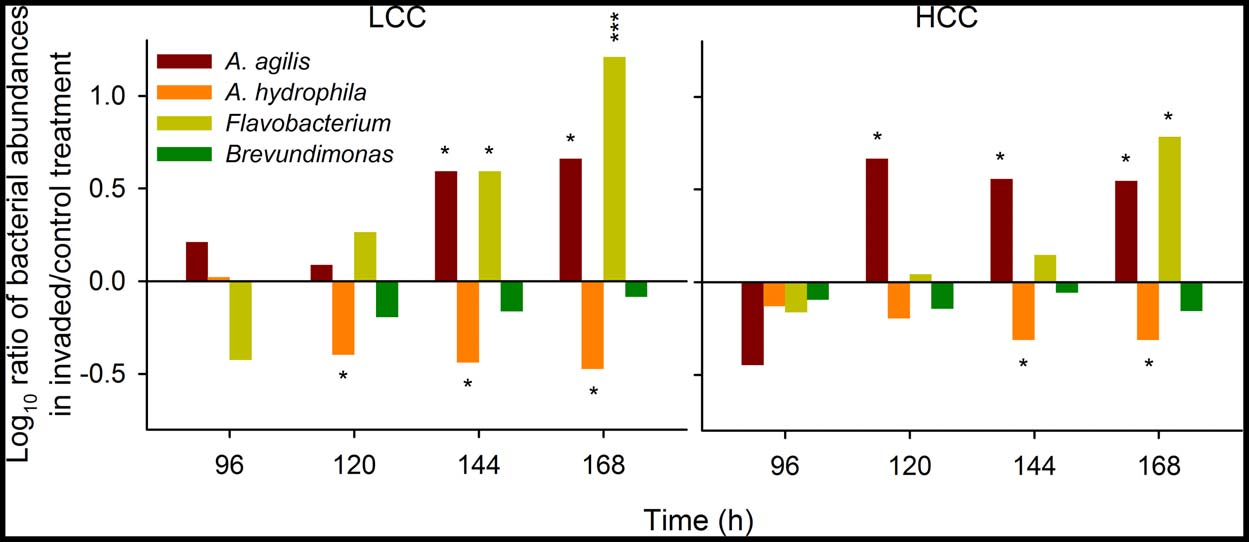
Relative success of bacteria. Numerical ratio of abundance of *A. agilis*, *A. hydrophila, Brevundimonas* sp. and *Flavobacterium* sp. achieved in invaded community compared to that in control community either in low- (LCC) or in high-carbon concentration (HCC) treatments. Asterisks above or below bars indicate significant differences in abundance of bacterial strains in the invaded community from that in the control community (two-way repeated measures ANOVA). * P<0.05, *** P<0.001.

**Figure 5:**
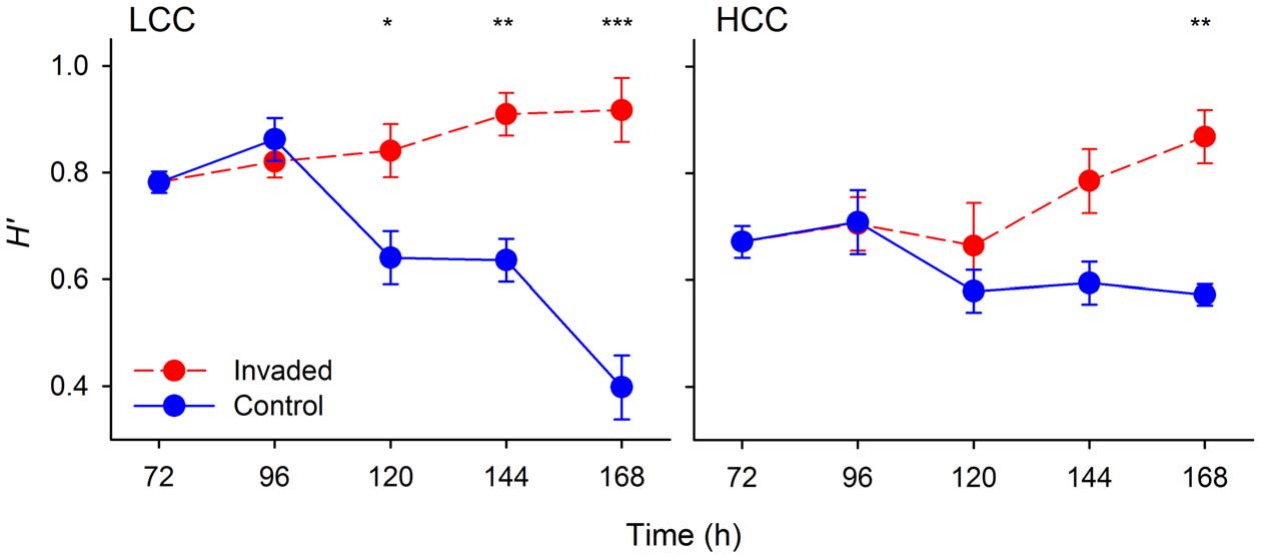
Community diversity. Shannon-Wiener (*H’*) diversity index in control and invaded communities. For consistency, *H’* in invaded communities was determined without regards of the relative contribution of *L. planktonicus*. Calculations were made both, for low- (LCC) and high-carbon concentration (HCC) treatments. Values are means of triplicates, error bars = SD. Asterisks above plots indicate significant differences in diversity index in the invaded community from that in the control community (two-way repeated measures ANOVA). * P<0.05, ** P<0.01, *** P<0.001.

Invasion by *L. planktonicus* significantly affected the composition of the bacterial communities (Figure 4). Abundances of *A. hydrophila* significantly declined after the invasion while those of *A. agilis* and *Flavobacterium* significantly increased over time as compared to control communities. Abundances of *Brevundimonas* were only slightly lower in the presence of the invader (Figure 4). The changes within the community composition were also significantly reflected in the relative species diversity (Shannon-Wiener index, Figure 5). The presence of *L. planktonicus* had a positive effect on the intrinsic species diversity of the resident community in both LCC and HCC treatments while the opposite pattern was found in the control (non-invaded) communities, particularly in the LCC treatment (Figure 5).

**Table 1:** Abundances of bacteria in different treatments at 168 h.

	LCC			HCC		
Bacteria	Pure culture	Community	Inv. community	Pure culture	Community	Inv. community
*A. agilis*	4.99±0.5	0.02±0.01	0.06±0.02	48.09±5.7	0.14±0.04	0.49±0.1
*A. hydrophila*	15.07±2.1	11.85±2.1	4±0.8	56.1±1.5	42.4±2.2	20.74±1.2
*Brevundimonas*	13.49±1.4	1.63±0.6	1.35±1.1	74.17±9.4	11.64±0.8	8.17±1.1
*Flavobacterium*	0.57±0.1	0.05±0.02	0.76±0.2	2.77±0.3	0.34±0.05	2.1±0.3
*L. planktonicus*	12.19±1.4	NA	8.62±1.1	35.32±1.3	NA	32.84±1.9

Abundances (10^6^ cells ml^−1^) of *A. agilis*, *A. hydrophila, Brevundimonas* sp., *Flavobacterium* sp. and *L. planktonicus* achieved at 168 h in pure culture, community and in invaded community either in low- (LCC) or in high-carbon concentration (HCC) treatments. Values are means of triplicates ± SD. NA – not available.

## Discussion

### Short-termed Effects of the Invasion

The impact of ecological invasions is generally described in terms of loss of resident species diversity or as changes in the physical and chemical properties of the invaded environment when applied to macroecology [35]. Researches having microbes as invaders usually focused on the potential spread of pathogens and on its direct impact on upper trophic levels [3]. As the relation between resources level and susceptibility to invasions of a system is still unclear [36], we tested the outcome of the invasion by a non-pathogenic bacterium on a simplified system at two different carbon concentration levels.

In natural aquatic environments, ecological variables and microbial community composition are never really steady, but every ecosystem’s equilibrium is determined by a succession of different and partly recurrent conditions, usually never lasting for more than a few days [37]. To appropriately mime these unstable conditions, we focused on the short-termed effects of the invasion: our artificially assembled resident communities developed only for a limited number of generations (8 to 40, depending on the bacterial species). Moreover, the invader was introduced into the resident community at its early stationary growth phase to examine the potential success of the invasion at the limited substrate availability. Then, we assessed the impact of the invasion over a similar amount of generations. Although our experiment performed under controlled laboratory conditions may be regarded as an extreme simplification of the natural ecosystem, such a setup is the most powerful tool nowadays available to examine possible species-specific interactions that are virtually inaccessible in complex natural systems [38].

### Bacterial Growth and Competition Patterns

In our experimental system, the non-invaded (control) community was dominated by *A. hydrophila* and partly by *Brevundimonas* (Figure 3A; Table 1). Contrary, abundances of *A. agilis* and *Flavobacterium* were extremely reduced with constantly decreasing numbers, suggesting their possible outcompeting if the given experimental conditions would continue a few days longer. This trend was observed at both selected substrate levels. The two “opportunistic“ species, i.e. *A. hydrophila* and *Brevundimonas*, supposed to win the competition for nutrients, dominated the communities, thus potentially confirming their general ecological success in waters [14]. On the other hand, the two species defined as “specialists” in our system, likely suffered from the absence of specific constellations such as protistan predation or algal blooms and therefore could not successfully compete within the community [39], [40].

Interestingly, comparing the abundance of bacteria in the control communities with that in pure cultures, the population size of *Brevundimonas*, despite being able to maintain a significant proportion in the community, was strongly reduced by competition with *A. hydrophila*. Even more drastic was the reduction of *A. agilis* (Figures 1 and 3A; Table 1). Overall, none of the bacterial species in the community could reach abundances comparable to those in pure culture (Table1), confirming a “cost of competition” scenario [41], that affected not only the less competitive strains, but also the dominating *A. hydrophila* (with a loss of about 30% in the control community). Moreover, total abundances in the communities were not higher than those of the best performing strain (*Brevundimonas,* Figures 1 and 2) which further supported the effects of inter-specific competition. Although *L. planktonicus* was added to the community already developing for 72 h, it rapidly reached abundances comparable to those in pure cultures (Figures 1 and 3B). This suggests that the invader efficiently utilized available resources and was least affected by the presence of the co-cultured strains although abundances of *L. planktonicus* in pure cultures were not higher than abundances of other strains (*Brevundimonas* and *A. hydrophila*).

In the control community, the condition of partial stability characterized by limitation in nutrients available, resulted in a continuous increment in the proportion of the most competitive species (*A. hydrophila*), especially under LCC conditions, and in a constant reduction in proportions of all other community members (Figure 3A), as already noticed for *Brevundimonas* and *A. agilis* also in pure cultures. This trend resulted in a strong decrement in diversity of the LCC community (Figure 5), while in HCC only a slight reduction of diversity could be detected.

### Invasion Impacts on Community Structure

The invasion by *L. planktonicus* had a significant impact on the temporal dynamics of the communities (Figure 4): irrespective of the nutrient content and growth rates in both LCC and HCC, abundances of *A. hydrophila* reduced by more than 50%. Thus, it appears conceivable that the invader underwent and rapidly won direct competition for resources with the dominant resident species with which it shares similar “opportunistic” ecological traits [14], [20]. However, constant abundances of *A. hydrophila* in the control community during its stagnant growth phase imply that resource competition was not the only factor responsible for a rapid decrease of its population size after the invasion. It seems likely that *L. planktonicus* actively inhibited growth and induced mortality of *A. hydrophila*, e.g. through the action of allelochemicals, as it has been demonstrated for *Pseudomonas aeruginosa* inhibiting *A. hydrophila* by quorum sensing-regulated production of secondary metabolites while competing for nutrients [25]. Nevertheless, the effects of secondary metabolites remained beyond the scope of our study and additional experiments would be required to specifically address the nature of this interaction. Our findings on the negative effects of *L. planktonicus* on *A. hydrophila* corroborated the earlier study where *L. planktonicus* suppressed growth of filamentous *Flectobacillus* sp. [42]. In contrast, *L. planktonicus* had a beneficial effect on growth of *Sphingobium* sp. [43]. Thus, a variety of species-specific interactions between *L. planktonicus* and other planktonic bacteria may be assumed.

Surprisingly, *A. agilis* and *Flavobacterium* benefited from the invasion: their abundances significantly increased in comparison to the control community, in LCC and HCC (Figure 4). The interaction between the invader and *A. hydrophila* likely temporarily facilitated the substrate availability for the “losing” resident species. Introduced bacterium likely possessing different resource use capabilities significantly modified the community functioning indirectly favouring bacteria formerly incapable of accessing the substrates as reported previously [10]. Moreover, allelopathic interactions between the invader and the resident species could promote their growth [44]. *Flavobacterium* as well as some members of the acI clade of *Actinobacteria* are known to readily utilize NAG [27], a principal component of peptidoglycans. Significantly higher abundances of *Flavobacterium* and *A. agilis* in the invaded community towards the end of the experiment (Figure 3) could be explained by the increased availability of NAG originating from the fragmentation of *A. hydrophila* cells. The latter phenomenon could be also responsible for the apparent lack of growth of *Flavobacterium* in pure culture (Figure 1).

In the presence of the invader, *Brevundimonas* displayed similar decreasing trends to *A. hydrophila* especially in LCC where it lost about two thirds of its population size (Figure 3B). In contrast to the latter strain, abundances of *Brevundimonas* dropped also in the control community towards the end of the experiment (Figure 3A). Thus, this decrease cannot be directly attributed to the impact of the invader but rather to low competitiveness of *Brevundimonas* within the community.

The invasion by *L. planktonicus* significantly altered the established structure of the community that was subsequently adjusting towards a new apparent steady state [45]. The ecological advantage of the “losing” species would most probably decrease with the adaptation of the new dominant species followed by an increasing stability of the system. However, in nature microbes are permanently subjected to rapidly changing environmental conditions that alter the competitive interactions within a new and repeatedly different environment [46]. Within this unstable equilibrium also periodical invasions by non-resident species can be considered a positive event in breaking stability, thus reducing the risk of outcompeting for the temporarily most disadvantaged species. In our experimental system the invasion by *L. planktonicus* was the key factor that allowed 2 of the 4 species of the assembled community not to be outcompeted. On the other hand, the invasion did not change the total abundances in the community, nor their temporal dynamics (Figure 2). The invader did not change the overall productivity of the community confirming thus the prediction of the model postulated by Loreau and Mouquet [47]. Deeper analyses are nevertheless required to assess an accurate relation between diversity and productivity, that is hard to define in an extremely simplified artificial system, due to other effects (e.g. dominance) highly affecting the community structure [48].

In contrast to previous studies suggesting that invasions occurring at intermediate immigration levels have the most pronounced impact on the resident community structure and functioning [9], [49], our experiment showed that the dispersal rate of 1% was beyond the threshold level required for the successful establishment of *L. planktonicus* within the community. This could be due to specific ecophysiological properties of *L. planktonicus* and/or due to simplicity of our experiment employing only four bacterial strains that could leave more niches available for the invader as compared to natural bacterial communities with much higher species richness. Our finding also suggests that *L. planktonicus* could be very successful in colonizing natural bacterial communities in new habitats.

This study also provided a clear evidence for the ability of *L. planktonicus* to shape an aquatic bacterial community in a few hours: the invader showed a rapid response to experimental manipulations and was overly competitive which is in agreement with previous studies using *L. planktonicus* in batch co-culture experiments [42], [43]. In a predators-free system, *L. planktonicus* numerically dominated the community and at the same time indirectly preserved the diversity. Moreover, this trend was observed independently of the two carbon concentration levels. Although no differences in species richness occurred between the control and invaded communities, the latter community could be characterized by a more even distribution among the species. Our results thus suggest the important role of *L. planktonicus* in structuring bacterial communities in lentic environments during periods of limited predation pressure.

To conclude, we could clearly demonstrate that a successful invasion by a non-resident species significantly modified the structure of the bacterial community: the invader suppressed the population size of the formerly dominating member of the community (*A. hydrophila*) which in turn had a positive effect on species diversity of the resident community. Hence, alternatively to the common view, our data indicate that periodical bacterial invasions, as other events such as predation, viral lysis or algal succession that modify the stability of a natural microbial system, may have an overall beneficial effect on the invaded community by opening new temporary ecological niches and thus indirectly support species diversity.
